# Case Report: Effective Treatment for Acute Chlorpyrifos Poisoning Complicated by a Non-ST-Segment Elevation Myocardial Infarction

**DOI:** 10.3389/fcvm.2021.623708

**Published:** 2021-04-30

**Authors:** Changqing Ye, Qiang Zhang, Yongsheng Chao, Chun Yin

**Affiliations:** ^1^Department of Cardiovascular Medicine, The 902nd Hospital of PLA Joint Service Support Force, Bengbu, China; ^2^Department of Physiology and Pathophysiology, Fourth Military Medical University, Xi'an, China

**Keywords:** acute myocardial infarction, chlorpyrifos poisoning, treatment strategy, atropine, antiplatelet agents

## Abstract

**Background:** Acute myocardial infarction (AMI) is a rare complication of acute organophosphorus pesticide poisoning. Although chlorpyrifos has been widely used as an organophosphate insecticide, a few cases of AMI complicated by chlorpyrifos poisoning have been reported thus far. Hence, a suitable treatment strategy remains to be explored.

**Case Presentation:** Based on the clinical manifestations, medical history, results of an auxiliary examination, and serum biomarkers, a 65-year-old male farmer with complaints of nausea, vomiting, chest tightness, and pain was clearly diagnosed as having a severe chlorpyrifos self-poisoning with acute non-ST-segment elevation MI. Because the patient and his family confirmedly refused a coronary intervention, conservative treatment was used instead. It should be noted that there were some conflicts of the management for chlorpyrifos poisoning and AMI. Although rapid atropinization would contribute to the relief of muscarinic symptoms, it would also lead to an increased heart rate and myocardial oxygen consumption in AMI. Furthermore, the reduction of platelet aggregation, which is necessary for coronary recanalization of an AMI patient, is known to aggravate the gastrointestinal injury caused by poisoning. In this case, these conflicts were properly addressed, which led to an excellent effect and prognosis of the patient.

**Conclusions:** To our knowledge, this is the first case report of acute chlorpyrifos poisoning with AMI. It is emphasized that patients with chest pain or coronary heart disease should be treated with atropine more cautiously because of the possible AMI. Moreover, proper resolution of conflicts in the management for chlorpyrifos poisoning and AMI played contributing roles in patient improvement.

## Introduction

Chlorpyrifos is a highly efficient, broad-spectrum, organophosphate insecticide that is widely used in agricultural production (1). Compared with traditional organophosphate insecticides, cholinesterase is inhibited more strongly and lastingly by chlorpyrifos. Besides, atropine poisoning is prone to occur during the treatment of chlorpyrifos poisoning (2). Acute myocardial infarction (AMI) is a rare complication of acute organophosphorus pesticide poisoning that has a high risk of mortality. To our knowledge, this is the first report of chlorpyrifos poisoning combined with AMI. In this report, the disease course and clinical manifestations are introduced in detail. Moreover, conflicts of treatment in chlorpyrifos poisoning and AMI are highlighted. Eventually, the patient showed a dramatic improvement after appropriate medical management.

## Case Description

A 65-year-old male farmer presented to our hospital with complaints of nausea, vomiting, chest tightness, and chest pain in March 2020. Three hours prior to presentation, the patient consumed 400 mL of chlorpyrifos in an attempt to commit suicide. Then, he presented with nausea, vomiting, sweating, and muscle tremors accompanied by slight chest tightness and pain. After he was found by his family, the patient was immediately sent to a local community hospital where gastric lavage was carried out and 10 mg of atropine was intravenously injected. However, the symptoms were not resolved, and his chest pain worsened. For further treatment, he was transferred to our hospital. His medical history included hypertension for 20 years and coronary heart disease for half a year. Six months ago, the patient underwent percutaneous coronary intervention (PCI), during which, a stent was implanted in the left anterior descending coronary artery. He consumed alcohol occasionally and had a 20-year history of smoking and smoked 40 cigarettes per day.

On physical examination, the patient was restless. His axillary temperature was 36.9°C, blood pressure was 145/100 mmHg, heart rate was 105 beats/min (bpm), and respiratory rate was 20 breaths/min. The diameters of both pupils were 4 mm. His heartbeat was fast and regular, without a murmur, and his upper abdomen was mildly tender. The skeletal muscle of the extremities went into spasm, with an increased muscle tone. The lungs were clear, and the neurologic examination was unremarkable.

**Figure 1:**
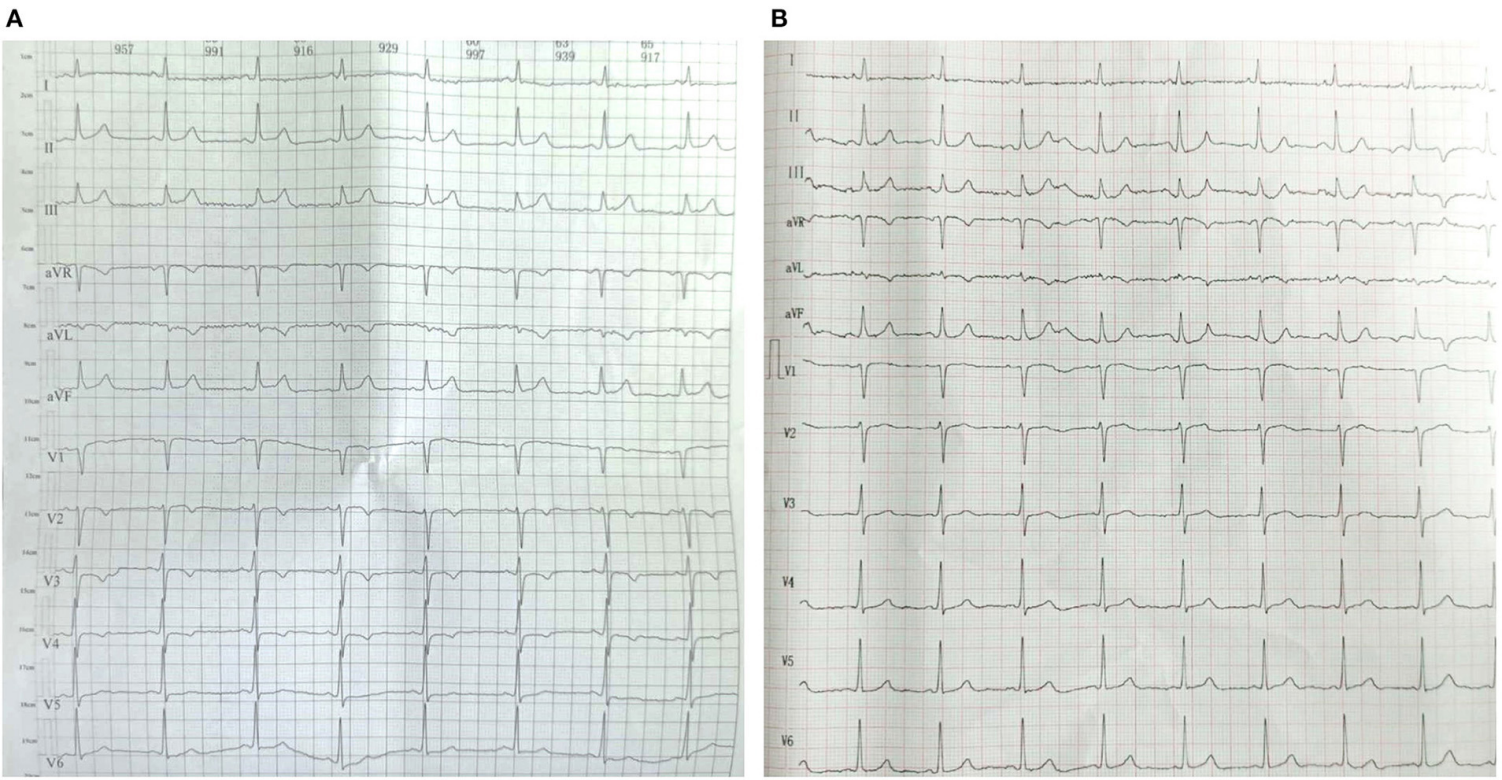
Electrocardiogram of the patient before admission **(A)** and at discharge **(B)**.

In the coagulation analysis, the international normalized ratio was 1.52, and the D-dimer level was 1.044 μg/mL. Cholinesterase level was 498 U/L, NT-pro BNP was 3,667.4 pg/mL, cardiac troponin I was 1.005 ng/mL, creatine kinase was 526 U/L, creatine kinase isoenzyme-MB was 34 U/L, alpha-hydroxybutyric dehydrogenase was 220 U/L, and lactate dehydrogenase was 282 U/L. Levels of electrolytes, as well as tests for liver and renal function, were within normal limits. As shown in Figure 1A, an electrocardiogram taken at the local community hospital showed a symmetrical negative T wave in leads V1–V6 and an upsloping ST-segment at the J point continuing into positive symmetrical T waves in leads II, III, and aVF. Chest radiography and abdominal ultrasound revealed no obvious abnormalities.

Based on the above clinical manifestations, medical history, result of auxiliary examinations, and level of serum biomarkers, the patient was conclusively diagnosed with an acute non-ST-segment elevation MI, severe chlorpyrifos poisoning, and grade 1 hypertension (very-high-risk group).

**Figure 2:**
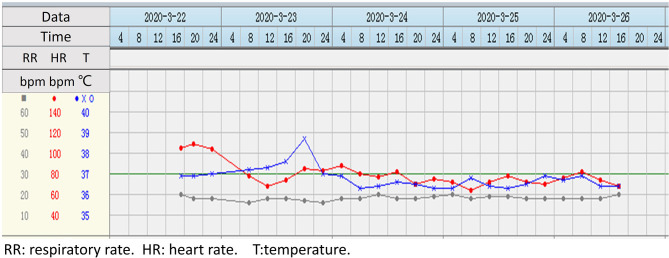
Real-time monitoring of the patient's vital signs during hospitalization.

After admission to the cardiac care unit, the patient received oxygen by nasal cannula. Because the patient and his family refused coronary intervention, pharmacologic treatment was provided instead. For AMI, the patient was orally administered with 180 mg of ticagrelor, 0.3 g of aspirin, 10 mg of rosuvastatin calcium, and 25 mg of metoprolol tartrate. For hypertension, oral valsartan (40 mg) in the form of dispersible tablets was given. For chlorpyrifos poisoning, he was treated using an intravenous injection of 2 g of pralidoxime iodide for the revitalization of cholinesterase. Additionally, parenteral nutrition support and 40 mg of pantoprazole sodium injection for rehydration, maintenance of nutrition, and protection of gastric mucosa were provided. On the following day, his chest pain diminished. However, he reported an obvious upper abdominal pain, accompanied by tarry stools. Considering that the bedside abdominal ultrasound revealed none of the clinically significant findings, he was treated with 8 mg of norepinephrine tartrate injection and 2 g of oral sucralfate tablets to control the gastrointestinal bleeding and protect the gastric mucosa. As shown in Figure 2, his heart rate dropped to 79 bpm after taking metoprolol tartrate tablets. A low dose of atropine and metoprolol tartrate was simultaneously adjusted to maintain the heart rate to <90 bpm. At night, he developed a fever of 38.6°C. A routine blood test revealed that his WBC count was 6,200 per cubic millimeter, with 73.9% of neutrophils, 14.1% of lymphocytes, and 12.0% of monocytes. His hemoglobin level was 12.8 g/dL. The patient was treated with an injection of 0.9 g of lysine acetylsalicylate and 3 g of cefoperazone sodium.

**Figure 3:**
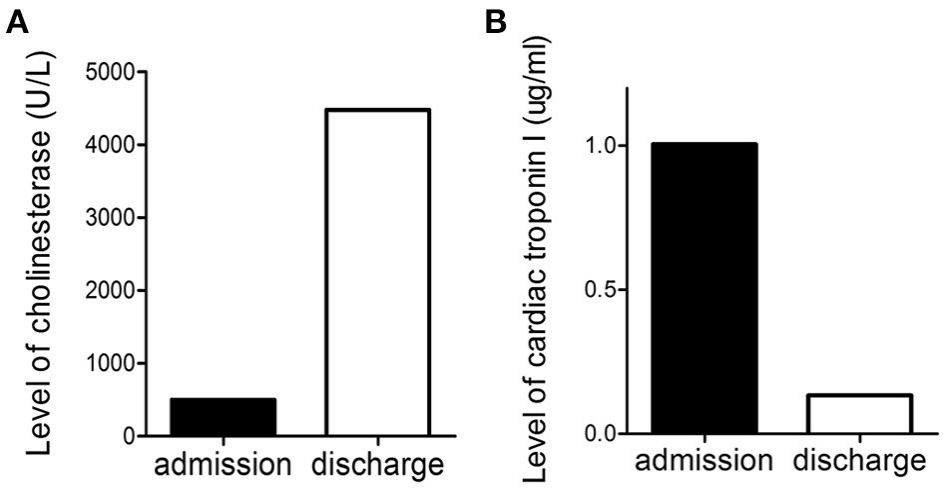
Level of cholinesterase **(A)** and cardiac troponin I **(B)** before admission and at discharge.

On day 4 of admission, his abdominal pain eased. Re-examination of the electrocardiogram revealed a normal sinus rhythm and the T-wave inversion in leads V1–V4 disappeared, without a pathological Q wave (Figure 1B). The patient was allowed to consume some liquid food, and the infusion volume was reduced. On day 5 of admission, cholinesterase levels rose from 498 to 4,476 U/L (Figure 3A), and cardiac troponin I decreased from 1.005 to 0.134 ng/mL (Figure 3B). Due to economic reasons, the patient and his family expressed a strong desire to be sent home for further rehabilitation. He continued taking aspirin, ticagrelor, and rosuvastatin after discharge. When followed-up on August 26, 2020, his condition was stable, and delayed encephalopathy was not observed.

## Discussion

Among patients with organophosphate poisoning, cardiac complications represented by myocardial injury (3), cardiac arrhythmias (4), and cardiac arrest (5) might be serious and fatal. AMI is a rarely reported complication of organophosphate poisoning. Two previous studies on 22,425 female and 55,748 male subjects explored the relationship between pesticide use and risk or mortality from AMI (6, 7). However, no case of AMI simultaneously accompanying organophosphate poisoning, especially chlorpyrifos, had been reported. In this case, AMI was clearly defined by increased levels of cardiac biomarkers together with either compatible symptoms or ECG changes. In a previous study, STEMI was diagnosed when the ST-segment elevation at the J point ≥1 mm was seen in at least two contiguous leads in any location on the ECG, new left bundle-branch block occurred, or documented new Q waves were observed. In the absence of these observation on the ECGs before admission and at discharge, the patient was considered to have a NSTEMI. The patient with AMI and poisoning was demonstrated for the first time. This is especially important for the field of this case provides valuable experience for the treatment of acute chlorpyrifos poisoning with AMI.

In this case, chest tightness and pain occurred after chlorpyrifos self-poisoning and worsened after gastric lavage and atropine injection. Some of the possible mechanisms for AMI are listed as follows. First, direct cardiotoxicity, sympathetic and parasympathetic overactivity, and hypoxemia resulting from chlorpyrifos poisoning played critical roles in the formation of AMI. Second, coronary artery spasm caused by pain and tension during gastric lavage could aggravate myocardial ischemia. Moreover, rescue atropine can increase the heart rate and myocardial oxygen consumption; hence, it is important to note that patients with chest pain or coronary heart disease should be treated with atropine more cautiously because of the possibility of an AMI.

Another highlight of this case is that we noted and addressed the conflicts of treatment for chlorpyrifos poisoning and AMI. First, there was no delay in atropinization, which contributed to the control of muscarinic symptoms. However, atropinization can increase the heart rate and myocardial oxygen consumption, which are harmful to patients with myocardial infarctions. To address this issue, beta-blockers and atropine were simultaneously administered to maintain the patient's heart rate to <90 bpm. Moreover, the reduction of platelet aggregation, which is necessary for coronary recanalization of AMI patients, would aggravate the gastrointestinal injury caused by poisoning. Synchronous use of an oral hemostatic agent and gastric mucosa protector proved to be an effective strategy. Although massive infusion would be necessary for fasting patients, it might also increase the risk of heart failure for AMI patients. Therefore, the volume of infusion should be decreased if the patient is able to ingest water and liquid food.

A limitation of the medical management reported herein was that, although we planned to perform PCI when the patient's condition stabilized, both he and his family resolutely refused the intervention citing economic reasons. Besides, cardiovascular magnetic resonance imaging failed to be performed due to the lack of a cardiac MRI machine in our hospital. Data of ejection fraction were unavailable.

In conclusion, this report provides insights into the characteristics and effective medical treatment strategy of chlorpyrifos poisoning with AMI.

## Data Availability Statement

The raw data supporting the conclusions of this article will be made available by the authors, without undue reservation.

## Ethics Statement

Written informed consent was obtained from the individual(s) for the publication of any potentially identifiable images or data included in this article.

## Author Contributions

QZ and YC analyzed and interpreted the patient data regarding the disease. CYe and CYi were major contributors in writing the manuscript. All authors read and approved the final manuscript.

## Conflict of Interest

The authors declare that the research was conducted in the absence of any commercial or financial relationships that could be construed as a potential conflict of interest.
